# From Plant Chemistry to Reproducible Antidiabetic Products: A Critical Review of Molecular Targets, Clinical Evidence, and Translational Gaps

**DOI:** 10.3390/molecules31172986

**Published:** 2026-08-26

**Authors:** Pirscoveanu Denisa Floriana Vasilica, Diana-Maria Trasca, Adina Maria Kamal, Renata Maria Varut, Daniela Cîrțînă, Romeo Popa, Pluta Ion Dorin, Dîrnu Rodica, Maria Stoica, Coancă-Staicu Cristina Teodora, George-Alin Stoica

**Affiliations:** 1 Department of Neurology, Faculty of Medicine, University of Medicine and Pharmacy of Craiova, 200349 Craiova, Romania; denisa.pirscoveanu@umfcv.ro; 2 Department of Internal Medicine, University of Medicine and Pharmacy of Craiova, 200349 Craiova, Romania; diana.trasca@umfcv.ro; 3 Research Methodology Department, Faculty of Pharmacy, University of Medicine and Pharmacy of Craiova, 200349 Craiova, Romania; coancastaicucristinateodora@gmail.com; 4 Faculty of Medical and Behavioral Sciences, Constantin Brâncuși University of Târgu Jiu, 210185 Targu Jiu, Romania; daniela.cirtina@e-ucb.ro (D.C.); dorin.pluta@e-ucb.ro (P.I.D.); rodica.dirnu@e-ucb.ro (D.R.); 5 Department of Pharmacology, University of Medicine and Pharmacy of Craiova, 200349 Craiova, Romania; romeo.popa@umfcv.ro; 6 Department of Intensive Care and Anesthesia, Emergency County Hospital, 200642 Craiova, Romania; maria.stoica@umfcv.ro; 7 Department of Pediatric Surgery, Faculty of Medicine, University of Medicine and Pharmacy of Craiova, 200349 Craiova, Romania; alin.stoica@umfcv.ro

**Keywords:** diabetes mellitus, medicinal plants, natural products, phytochemicals, botanical drugs, pharmacokinetics, herb–drug interactions, diabetic complications

## Abstract

Diabetes mellitus results from insulin resistance, progressive pancreatic β-cell dysfunction, dysregulated hepatic and adipose metabolism, oxidative stress, and inflammation. Plant-derived compounds can modulate several of these processes, yet pharmacological breadth does not necessarily produce a reproducible therapy. This critical narrative review links phytochemical identity and product composition to intestinal carbohydrate digestion, insulin secretion, hepatic glucose production, GLUT4 trafficking, AMPK and PPARγ signaling, the incretin–DPP-4 axis, renal glucose handling, and diabetic organ injury. Alkaloids, flavonoids, phenolic acids, tannins, saponins, and polysaccharides are considered alongside evidence concerning nephropathy, neuropathy, ocular disease, hepatopathy, and cardiomyopathy. Controlled human studies provide product-specific signals, particularly for chemically defined berberine preparations and mulberry alkaloids, whereas most other interventions remain supported by small, short, or chemically undercharacterized trials. The strongest candidates are those for which defined chemistry, plausible exposure, mechanism, and controlled clinical findings converge. Progress requires authenticated raw material, validated analytical fingerprints, pharmacokinetic and toxicological characterization, herb–drug interaction testing, and trials using the same standardized product. These interventions should remain supervised adjuncts or drug-discovery leads rather than substitutes for established diabetes treatment.

## 1. Introduction

Diabetes mellitus comprises a group of chronic metabolic disorders characterized by persistent hyperglycemia resulting from impaired insulin secretion, impaired insulin action, or both. Although hyperglycemia defines the disease clinically, diabetes is not simply a disorder of circulating glucose. In type 2 diabetes mellitus, insulin resistance in skeletal muscle, liver, and adipose tissue is initially compensated by increased pancreatic insulin secretion. As β-cell reserve declines, compensation becomes inadequate and glycemic control deteriorates. In type 1 diabetes, immune-mediated β-cell destruction produces an absolute insulin deficit. Across both major forms, prolonged exposure to excess glucose promotes oxidative stress, non-enzymatic glycation, endothelial dysfunction, inflammation, mitochondrial injury, and maladaptive activation of several metabolic pathways. These processes link metabolic dysregulation to progressive damage in the kidney, peripheral and autonomic nerves, retina and lens, liver, and cardiovascular system.

Contemporary diabetes management uses lifestyle intervention together with agents that increase insulin availability, improve insulin sensitivity, delay carbohydrate absorption, enhance incretin action, increase urinary glucose excretion, or replace insulin. These approaches have transformed prognosis, yet clinically important limitations remain. Diabetes is progressive in many patients, therapeutic responses vary, polypharmacy is common, and hypoglycemia or organ-specific adverse effects may constrain treatment. Access and affordability also differ substantially across regions. These realities help explain the continuing use of herbal medicines and the scientific interest in plant-derived compounds. The appropriate question is not whether natural products should replace established therapy, but whether chemically defined plant preparations or isolated phytochemicals can safely complement current treatment or serve as templates for new drugs.

Medicinal plants have been used for diabetes across Africa, Asia, the Middle East, Europe, Australia, and the Americas. The literature identifies approximately 350 plants used in traditional antidiabetic practice and describes leaves, bark, roots, rhizomes, bulbs, stems, flowers, seeds, fruits, and fruit pulp administered as extracts, decoctions, powders, or complex herbal mixtures [1,2,3,4]. This extensive and geographically diverse record provides a valuable discovery platform. Repeated selection of the same genera in unrelated medical traditions may indicate reproducible biological effects. Nevertheless, ethnomedical continuity is not equivalent to clinical efficacy, and traditional preparations often differ markedly from standardized products used in experimental research.

Plant-based therapy is pharmacologically interesting because it spans chemical space that is only partly represented in conventional compound libraries. Alkaloids, flavonoids, glycosides, terpenoids, peptides, lipids, tannins, saponins, anthocyanins, and polysaccharides have all been associated with glucose lowering [5,6]. Reports of nearly 200 isolated compounds and more than 108 active species from 56 families suggest a productive discovery field, but they also expose a classification problem: activity assigned to a species may arise from different organs, extraction procedures, or constituents. A useful review must therefore retain the identity of the tested preparation rather than treating the plant name as the intervention.

Primary antidiabetic activity refers to the direct improvement of glucose homeostasis through effects on insulin-producing cells and insulin-responsive tissues. Relevant mechanisms include the inhibition of intestinal glucose absorption; inhibition of α-amylase, α-glucosidase, and β-galactosidase; stimulation of insulin synthesis or release; inhibition of insulin degradation; protection or regeneration of pancreatic β-cells; increased glycogenesis and glycolysis; suppression of hepatic gluconeogenesis; increased glucose uptake through GLUT4 translocation; activation of AMPK or PPARγ; modulation of adiponectin; and enhancement of incretin signaling through DPP-4 inhibition [7,8,9,10]. These mechanisms overlap with validated pharmacological strategies and provide biologically plausible routes through which plant products may affect fasting and postprandial glycemia.

Secondary antidiabetic activity concerns the prevention or attenuation of chronic complications. Oxidative stress and inflammation are recurring targets because they participate in β-cell failure, endothelial injury, renal fibrosis, neuronal dysfunction, cataract formation, retinal damage, hepatic injury, and diabetic cardiomyopathy. Polyphenols, flavonoids, tannins, and related constituents may interrupt lipid-peroxidation chains, chelate transition metals, increase endogenous antioxidant enzymes, reduce polyol accumulation, inhibit aldose reductase, and limit apoptosis [8,11,12]. This distinction between glucose lowering and organ protection is critical: an extract may have limited acute hypoglycemic activity yet influence complication-related pathways, whereas a strong glucose-lowering effect in an animal model does not establish long-term human organ protection.

Interpretation must also consider the hierarchy of evidence. Enzyme assays and molecular docking can identify potential interactions but do not establish absorption or in-vivo exposure. Cell systems clarify signaling pathways but cannot reproduce whole-organism metabolism. Alloxan- and streptozotocin-induced models provide useful information on insulin deficiency, oxidative damage, biochemical markers, and histology, although they represent only selected aspects of human diabetes. Clinical observations and trials offer greater translational value, but their reliability depends on randomization, blinding, comparator selection, sample size, duration, standardized preparations, and the control of concomitant therapy. In the evidence base, preclinical evidence predominates; this must temper claims of clinical effectiveness.

Safety and quality are inseparable from efficacy. A botanical name alone does not define an intervention. Species authentication, chemotype, cultivation conditions, harvested organ, processing, solvent, extraction ratio, storage, and marker-compound content can all alter activity. Contamination, adulteration, or substitution may introduce additional risks. Plant products capable of lowering glucose may potentiate insulin or oral antidiabetic agents and cause hypoglycemia. Hepatic, renal, cardiovascular, allergic, and reproductive toxicities also require systematic assessment. Accordingly, clinical development should include pharmacognostic authentication, phytochemical profiling, contaminant limits, stability testing, dose–response characterization, toxicology, pharmacokinetics, and interaction studies.

**Figure 1:**
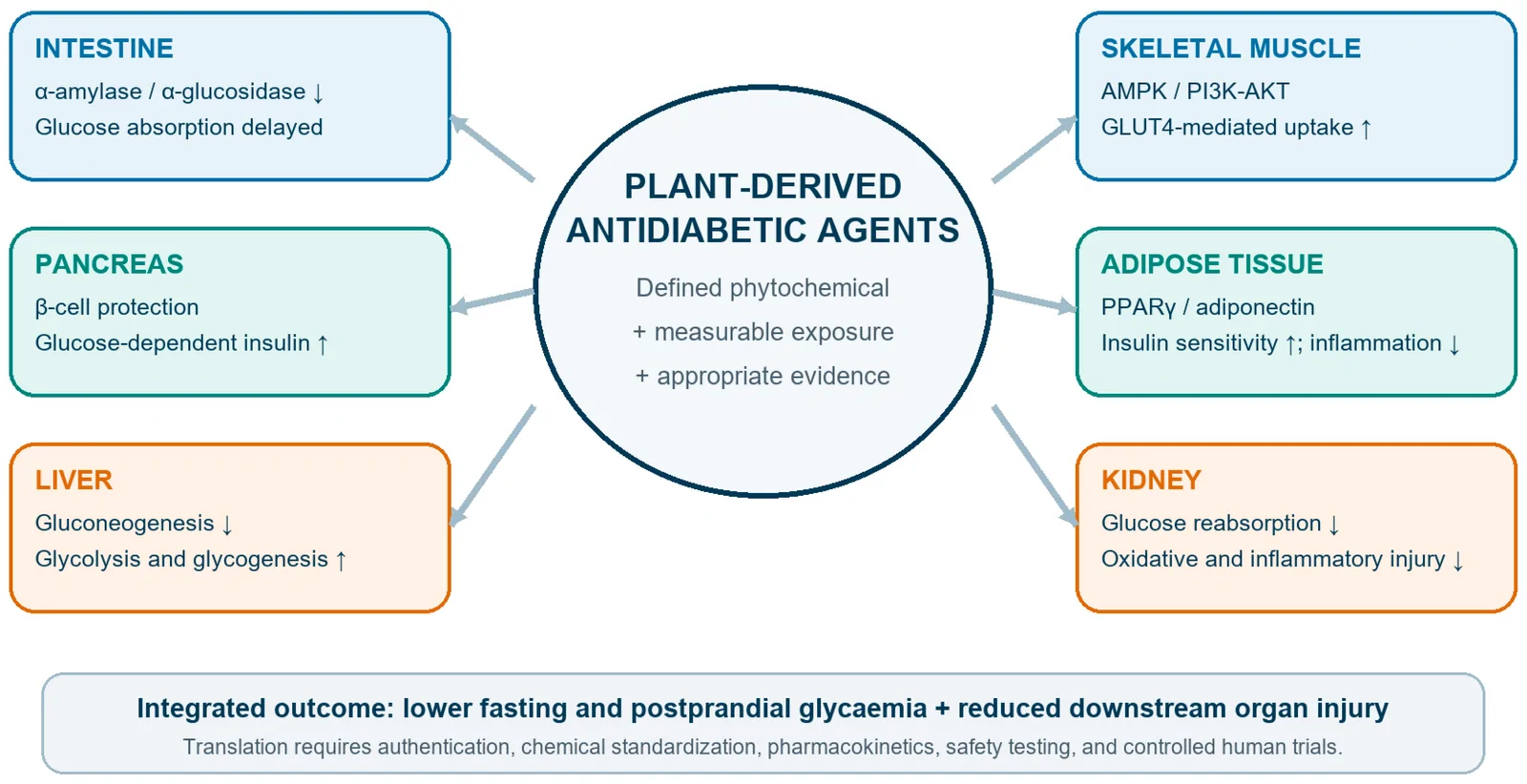
Conceptual multi-organ framework for plant-derived antidiabetic agents. Chemically defined plant preparations may act on intestinal carbohydrate digestion, pancreatic β-cell function, hepatic glucose production, muscle and adipose glucose uptake, and downstream renal, neural, ocular, hepatic, and cardiovascular injury. The figure is conceptual and does not imply equivalent evidence across organs. Created in BioRender. Varut R. (2026) https://app.biorender.com/illustrations/canvas-beta/670666effff8cafd86571cff.

This review examines plant-derived antidiabetic agents through a pharmaceutical-development lens. Rather than compiling species solely according to traditional use or glucose-lowering activity, it links botanical identity and phytochemical composition to molecular targets, tissue-level effects, human evidence, safety, and developability. The central contribution is an integrated chain—phytochemical identity → molecular target → organ response → clinical evidence → reproducible pharmaceutical product—that exposes where apparently promising candidates lose support during translation. Figure 1 summarizes this multi-organ framework, and the organ-specific evidence is consolidated in a single section with explicit separation of preclinical observations from controlled human findings.

## 2. Literature Search and Evidence Synthesis

This work was designed as a critical narrative review rather than a systematic review or meta-analysis. Historical, phytochemical, mechanistic, and complication-oriented studies were identified from primary reports and backward citation searching of key reviews. A targeted PubMed update was last performed on 23 July 2026. The core query combined (“diabetes mellitus” OR “type 2 diabetes”) with (“medicinal plant” OR phytotherapy OR phytochemical OR “natural product”); supplementary searches paired priority species or compounds with randomized trial, pharmacokinetics, bioavailability, safety, toxicity, or herb–drug interaction terms.

Eligible evidence included studies that identified the botanical species or compound and reported a relevant molecular, metabolic, organ-specific, safety, pharmacokinetic, or clinical outcome. Human studies were prioritized when product, dose, duration, comparator, background therapy, and clinically relevant outcomes could be identified. Mechanistic and preclinical studies were retained when they clarified a defined target, active constituent, tissue response, exposure constraint, or safety concern. Reports lacking intervention identity or interpretable outcomes were not used to support efficacy claims. Different plant organs, solvents, enriched fractions, isolated compounds, and proprietary formulations were treated as distinct interventions.

Evidence was classified as biochemical or cellular, animal, observational human, or controlled human evidence. Clinical studies were appraised qualitatively for randomization, blinding, comparator, sample size, attrition, duration, product characterization, multiplicity of outcomes, adverse-event reporting, and funding or commercial involvement. Priority was assigned when chemical definition, mechanistic coherence, exposure plausibility, and clinically interpretable human outcomes converged. No formal risk-of-bias instrument, exhaustive database search, duplicate screening procedure, or pooled effect estimate was applied; consequently, this targeted narrative synthesis cannot support a quantitative meta-analytic conclusion, and omission of a study should not be interpreted as evidence of ineffectiveness.

## 3. Global Ethnopharmacological Context

The geographical record is useful as an indicator of repeated ethnomedical selection, although recurrence across countries cannot establish efficacy. Frequently reported taxa include *Allium cepa*, *Allium sativum*, *Aloe vera*, *Azadirachta indica*, *Momordica charantia*, *Ocimum sanctum*, *Psidium guajava*, *Syzygium cumini*, *Trigonella foenum-graecum*, and *Zingiber officinale*. The same species may be prepared from different organs, extracted with different solvents, or administered at markedly different doses. Geographical convergence is therefore best used to prioritize candidates for comparative chemical and pharmacological study rather than as a surrogate for clinical validation [1,2,3,4].

## 4. Mechanistic Framework for Antidiabetic Activity

Antidiabetic plants cannot be understood through a single pharmacological model. Some act predominantly within the intestinal lumen, whereas others have been linked to pancreatic secretion, hepatic glucose output, peripheral uptake, adipose signaling, or protection from oxidative injury. Table 1 places these proposed actions alongside representative plants and compounds, allowing their points of convergence to be compared.

Inhibition of carbohydrate digestion is among the most immediately testable mechanisms. α-Amylase hydrolyzes complex starches, while intestinal α-glucosidases release absorbable monosaccharides. Partial inhibition can blunt postprandial glucose excursions and reduce the insulin demand created by carbohydrate-rich meals. Plant fibers may further slow gastric emptying or physically interfere with carbohydrate absorption [10,12]. Enzyme inhibition observed in vitro should nevertheless be interpreted in relation to potency, intestinal exposure, selectivity, and gastrointestinal tolerability. Very strong non-selective inhibition may produce effects analogous to pharmacological enzyme inhibitors, including fermentation-related discomfort.

Insulinotropic activity can arise from the closure of β-cell potassium channels, changes in calcium handling, increased expression or processing of insulin, or protection against oxidative impairment of stimulus–secretion coupling. Inhibition of insulin degradation and provision of calcium, zinc, magnesium, manganese, and copper may also support β-cell physiology [10]. Because insulin release requires residual functional β-cell mass, this mechanism is more relevant to insulin-resistant or early type 2 diabetes than to absolute insulin deficiency. Experimental reports should distinguish glucose-dependent secretion from indiscriminate secretagogue activity, which may increase hypoglycemia risk.

**Figure 2:**
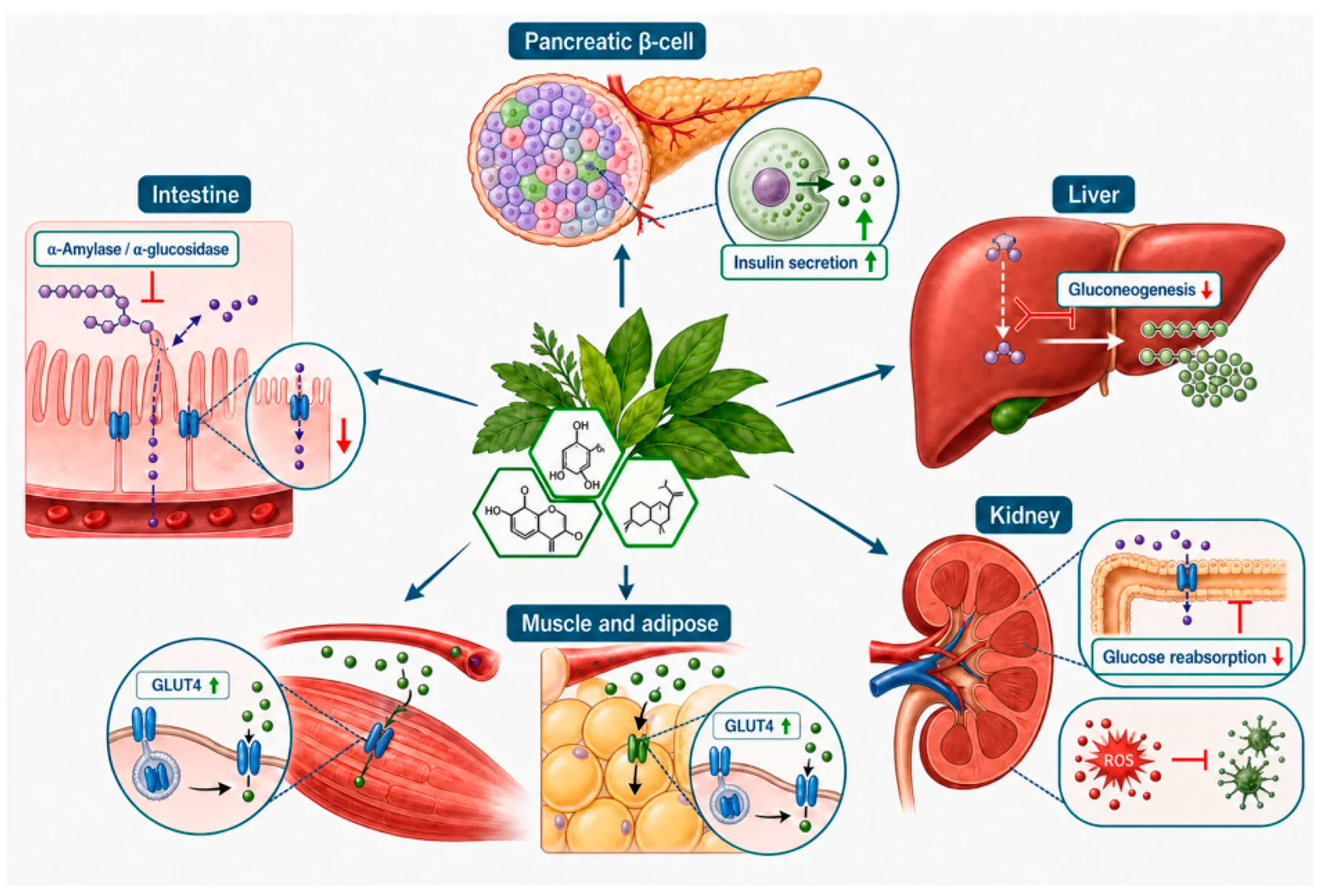
Multi-organ mechanisms of plant-derived antidiabetic agents. Plant-derived compounds may reduce intestinal carbohydrate digestion, support pancreatic β-cell function and insulin secretion, suppress hepatic gluconeogenesis, enhance GLUT4-mediated glucose uptake in muscle and adipose tissue, and reduce renal glucose reabsorption and oxidative stress. Created in BioRender. Varut R. (2026) https://app.biorender.com/illustrations/canvas-beta/6a62371858a31bddbdfd416f.

Hepatic glucose output is another central target. Increased hexokinase and glucokinase activity promotes phosphorylation and the utilization of glucose, whereas reduced glucose-6-phosphatase activity can limit hepatic glucose release. Enhanced glycogenesis directs glucose toward storage, and suppression of gluconeogenic pathways reduces fasting hyperglycemia. Several plant interventions in the evidence base altered these enzymes, although enzyme activity alone does not establish net metabolic flux. Isotope-tracer studies, tissue-specific signaling analysis, and metabolomics would strengthen causal interpretation. The relationships among intestinal carbohydrate digestion, pancreatic β-cell function, hepatic glucose production, glucose uptake in muscle and adipose tissue, and renal glucose handling are integrated in Figure 2.

## 5. Phytochemical Classes

From a pharmaceutical perspective, priority should be given to constituents that can be measured, linked to a plausible target, and followed through pharmacokinetic and clinical development. Table 2 compares selected lead compounds and marker groups according to their botanical origin, proposed actions, analytical value, and principal development constraint.

Chemical classification provides a more informative bridge between botanical identity and pharmacological behavior. Closely related molecules may differ in solubility, metabolism, target affinity, and tissue exposure, while constituents from unrelated classes may converge on the same pathway. Table 3 summarizes the major phytochemical groups discussed in this review and the activities most often associated with them.

Flavonoids form one of the largest and most intensively discussed groups in the literature. Their common C6–C3–C6 scaffold is modified through hydroxylation, methylation, glycosylation, and conjugation, producing major subclasses such as flavones, flavonols, flavanones, flavan-3-ols, anthocyanidins, and isoflavones [21,31]. These structural differences influence solubility, intestinal metabolism, protein binding, and access to intracellular targets. Consequently, results obtained with quercetin cannot automatically be attributed to rutin, kaempferol, luteolin, or a flavonoid-rich extract, despite overlapping antioxidant properties.

The antioxidant description of polyphenols should not be restricted to direct radical scavenging. At physiological concentrations, indirect regulation of Nrf2-related defense, inflammatory transcription factors, mitochondrial function, endothelial signaling, and kinase pathways may be more important than stoichiometric neutralization of radicals. Quercetin, for example, is described as scavenging reactive oxygen species, preventing lipid peroxidation, and chelating metal ions [36]. Baicalein and other flavonoids additionally influence glial and neuronal survival pathways [43,58]. Such pleiotropy is pharmacologically attractive but makes dose–target relationships difficult to define.

Alkaloids and saponins contribute a different chemical and pharmacodynamic profile. Berberine and related protoberberines are frequently linked to glucose uptake and metabolic signaling, whereas saponin-rich fractions are associated with hypoglycemic activity in several models. Polysaccharides may act through antioxidant, immune, microbiome, or barrier-related mechanisms rather than classical receptor binding. Future comparisons should therefore stratify interventions by chemical class and exposure instead of combining all plant products under a single phytotherapy category.

## 6. Plants and Compounds for Glycemic Control

Evidence for glucose lowering spans crude powders, aqueous or alcoholic extracts, enriched fractions, and isolated constituents. These preparations are not interchangeable. To make the hierarchy explicit, this section proceeds from controlled human signals to convincing preclinical evidence and then to conflicting or insufficient evidence; Table 4 retains the exact preparation and model for each representative intervention.

### 6.1. Controlled Human Evidence and Clinically Relevant Signals

Product-specific human signals have been reported for onion juice and historical *Pterocarpus* preparations, but these observations are not equivalent to contemporary randomized evidence.

**Table 4:** Representative plants investigated for glucose lowering, with preparation-specific evidence levels.

Plant	Preparation/Compound	Reported Effect and Mechanism	Evidence Level (Preclinical/Human)	References
*Aloe barbadensis* Mill.	Leaf extract; bitter principle	Chronic administration lowered glucose in alloxan-diabetic rats; proposed stimulation of insulin synthesis/release	Animal	[13]
*Artemisia dracunculus* L.	Extract; ethanolic extract tarralin	Bioenhancer increased activity; tarralin reduced glucose by 24% and affected glucagon-peptide receptor binding	Animal/in vitro	[59,60]
*Azadirachta indica* A. Juss.	Powder, aqueous and alcoholic extracts	Hypoglycemia in normal and diabetic animals; chronic treatment associated with β-cell regeneration and increased glycogen synthesis	Animal/limited human observation	[16,61]
*Catharanthus roseus*	Methanolic leaf extract	Marked glucose reduction in alloxan-diabetic rats; reported as greater than glibenclamide/metformin in the cited model	Animal	[3]
*Allium cepa*	Onion powder fractions; S-methyl cysteine sulfoxide; juice	Antihyperglycemic, antioxidant and hypolipidemic effects; normalization of hepatic enzymes; reduced postprandial glucose	Animal/human observation	[14,15,62]
*Berberis vulgaris* L.	Aqueous extract and saponins	Significant hypoglycemic activity in streptozotocin-diabetic rats; lipid effects also reported	Animal	[3,50]
*Momordica charantia*	Fruit/plant preparations	Frequently reported in ethnomedicine and experimental type 2 diabetes research	Preclinical/clinical literature	[6,11]
*Trigonella foenum-graecum*	Seeds and preparations	Glucose-lowering and complication-related effects; aldose-reductase inhibition discussed for cataract protection	Preclinical/clinical literature	[6,11,63]
*Gymnema sylvestre*	Leaf preparations	Recognized among widely used medicinal plants for diabetes	Preclinical/clinical literature	[11]
*Pterocarpus marsupium*	Bark extract; (−)-epicatechin	Lowered glucose, improved tolerance, promoted insulin release and β-cell repair in animal studies	Animal/historical human observation	[20]

### 6.2. Convincing Preclinical Evidence

The most coherent preclinical evidence links a chemically or botanically defined intervention to more than one relevant endpoint. *Aloe* and neem have been associated with insulin secretion or β-cell preservation, onion fractions with glucose, lipid, antioxidant, and hepatic-enzyme effects, and *Pterocarpus* bark or (−)-epicatechin with insulin release and experimental β-cell repair. These findings support target validation, but they do not establish clinical efficacy because model, dose, exposure, and preparation differ from human use [64,65].

### 6.3. Conflicting or Insufficient Evidence

Evidence remains insufficient when activity depends on a single acute model, a poorly characterized extract, an uncontrolled clinical observation, or a comparison that lacks exposure and safety context. Such candidates should be described as hypothesis-generating until the intervention is standardized, concentrations are shown to be pharmacokinetically plausible, and the result is independently replicated.

## 7. Pancreatic β-Cell Protection, Insulin Sensitivity, and Glucose Transport

β-Cell preservation is particularly attractive because progressive loss of functional β-cell mass drives worsening glycemia. Antioxidant flavonoids may reduce reactive oxygen species, lipid peroxidation, inflammatory signaling, and apoptosis, while selected extracts are reported to stimulate proliferation or regeneration. Evidence of histological regeneration after Azadirachta indica and functional effects of Aloe or Pterocarpus should be interpreted as preclinical signals rather than proof of human β-cell restoration [13,16,20].

In insulin-responsive tissues, enhanced GLUT4 translocation increases glucose uptake into skeletal muscle and adipose tissue. The literature links flavonoids and several extracts to PI3K/AKT and AMPK signaling and describes PPARγ activation, adiponectin release, incretin-mimetic activity, and DPP-4 inhibition as complementary routes for improving insulin sensitivity. These mechanisms are biologically plausible but require confirmation at clinically achievable exposures [21,22].

PPARγ regulates adipocyte differentiation, lipid storage, adipokine production, and systemic insulin sensitivity. Partial or context-dependent activation by plant constituents may theoretically improve glucose disposal without reproducing the full adverse-effect profile of potent synthetic agonists, although this remains to be demonstrated. Adiponectin provides a related link between adipose tissue and insulin-responsive organs: increased release can enhance fatty-acid oxidation and insulin sensitivity. Measurements of receptor activation or circulating adiponectin should be connected to tissue-specific glucose uptake and whole-body metabolic outcomes.

The incretin–DPP-4 axis offers another translational bridge to established pharmacology. Incretins enhance glucose-dependent insulin secretion, while DPP-4 terminates incretin action. Plant compounds that inhibit DPP-4 or mimic incretin effects could therefore lower glucose with a theoretically lower risk of glucose-independent insulin release. However, in vitro DPP-4 inhibition is meaningful only when active compounds reach sufficient systemic or intestinal concentrations and do not interfere non-selectively with related peptidases.

## 8. Oxidative Stress and Chronic Diabetic Complications

Oxidative stress is a cross-cutting mechanism rather than a single complication. Hyperglycemia increases mitochondrial reactive oxygen species, promotes lipid peroxidation, exhausts glutathione-dependent defense, and amplifies inflammatory and apoptotic signaling. Plant-derived antioxidants may directly scavenge radicals or indirectly increase superoxide dismutase, catalase, glutathione peroxidase, glutathione-S-transferase, and reduced glutathione. Because antioxidant effects are frequently measured together with lower glucose, studies should distinguish direct tissue protection from consequences of improved glycemia. Combination experiments with *Piper nigrum* and *Vinca rosea* provide an additional historical example of antioxidant-associated protection in alloxan-induced diabetes, although they do not identify the active constituent or establish clinical efficacy [66].

The polyol pathway is particularly relevant to tissues in which glucose uptake is not fully insulin dependent. Aldose reductase converts glucose to sorbitol, consumes NADPH, and thereby contributes to osmotic stress and impaired antioxidant recycling. Sorbitol accumulation and related redox disturbances have been implicated in lens, retinal, neural, and renal injury. Flavonoids, tannoid constituents, and selected plant extracts that inhibit aldose reductase therefore provide a plausible mechanistic bridge across several diabetic complications [44,45,63,67,68,69]. Experimental reversal of early peripheral-nerve dysfunction with aldose-reductase inhibition further supports the pathway while remaining distinct from proof of botanical efficacy [70,71].

**Figure 3:**
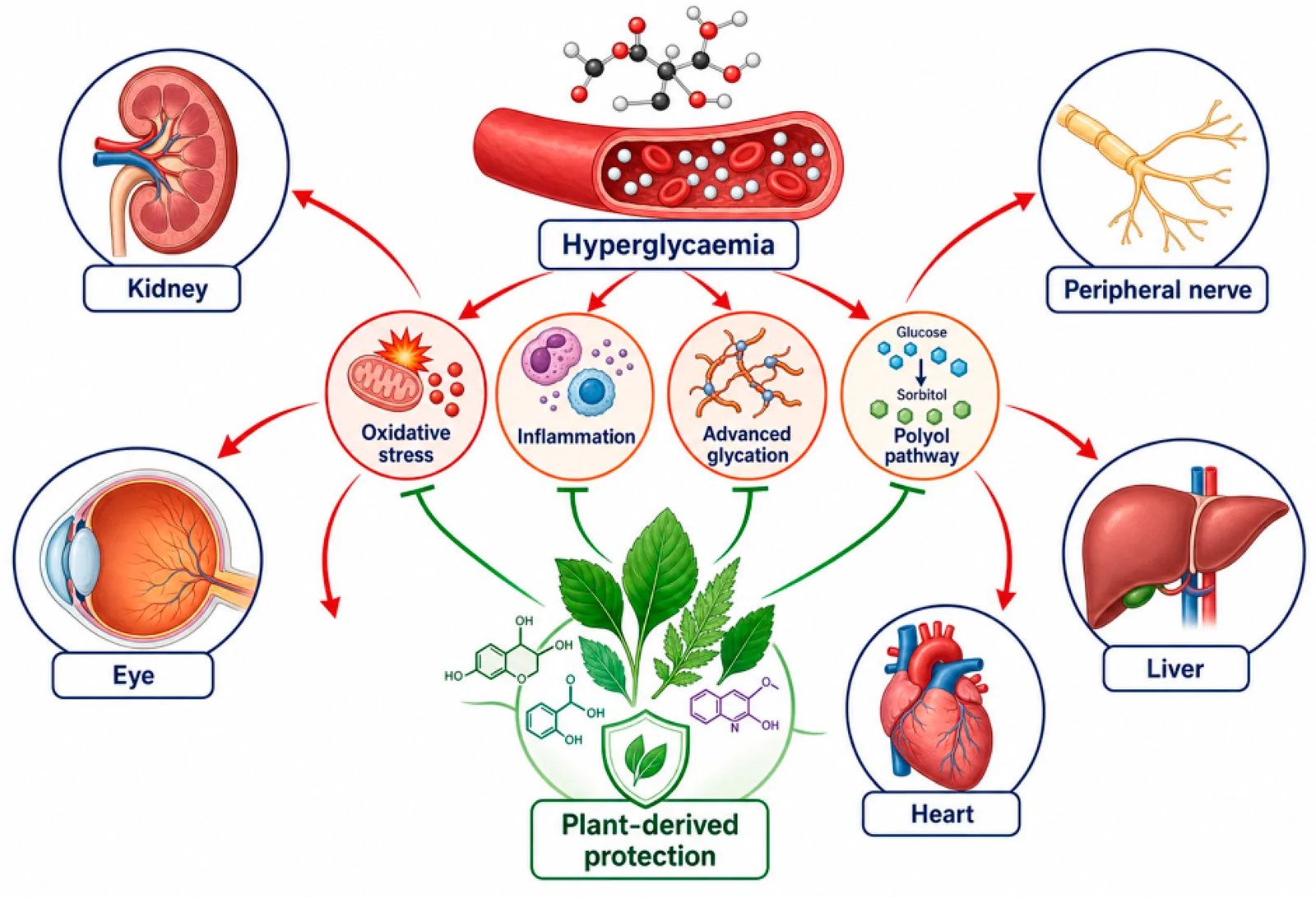
Shared pathological pathways targeted by plant-derived compounds in chronic diabetic complications. Hyperglycemia promotes oxidative stress, inflammation, advanced glycation, and polyol-pathway activation, which contribute to injury of the kidney, peripheral nerves, eye, liver, and heart. Plant-derived constituents may attenuate several of these interconnected pathways. Created in BioRender. Varut R. (2026) https://app.biorender.com/illustrations/canvas-beta/6a623aa8afe761dc6f20372a.

Advanced glycation and inflammatory signaling form additional bridges between metabolic exposure and tissue injury. Glycated proteins can alter extracellular-matrix properties, receptor signaling, vascular permeability, and immune activation. Antiglycation effects, metal chelation, radical scavenging, and preservation of endogenous antioxidants may act cooperatively. Nevertheless, biomarkers such as malondialdehyde or total antioxidant capacity are surrogate outcomes; robust organ protection should be demonstrated through functional and structural endpoints. These shared mechanisms and their links to renal, neural, ocular, hepatic, and cardiac injury are summarized in Figure 3.

## 9. Organ-Specific Effects

Evidence across diabetic complications is predominantly preclinical. Cellular or biochemical findings indicate mechanism; animal findings support tissue-level plausibility; controlled human outcomes are required for clinical efficacy. The following subsections retain this distinction for each organ system.

### 9.1. Diabetic Nephropathy

Renal benefit has usually been inferred from changes in biochemical or histological markers rather than from the long-term preservation of filtration. It is therefore important to distinguish direct renal actions from improvement secondary to lower glucose or blood pressure. The principal plants, preparations, and renal observations identified in the literature are compared in Table 5.

Experimental nephropathy studies predominantly support antioxidant and anti-inflammatory protection rather than a single glucose-lowering mechanism. Clinically meaningful translation requires prespecified renal endpoints—albuminuria, estimated glomerular filtration rate, histological fibrosis, and validated biomarkers—together with active surveillance for intrinsic nephrotoxicity. Representative compound-level evidence is summarized in Table 5.

### 9.2. Diabetic Neuropathy

The neuropathy literature is mechanistically diverse, encompassing oxidative stress, microglial activation, mitochondrial dysfunction, apoptosis, and impaired neuronal growth. Several studies also include functional measures such as nerve conduction, thermal sensitivity, or tactile allodynia. Table 6 relates the main plant-derived candidates to these experimental outcomes and indicates the evidentiary setting in which each was studied.

### 9.3. Diabetic Ocular Complications

Ocular complications provide one of the clearest examples of pathway-oriented phytopharmacology. Much of the evidence focuses on aldose reductase, sorbitol accumulation, protein glycation, glutathione depletion, and lens oxidative injury, although retinal inflammation and vascular damage are also represented. Table 7 organizes the main candidates by ocular target rather than by plant name alone.

The ocular literature is dominated by cataract models and inhibition of the polyol pathway. Aldose reductase converts glucose to sorbitol; excessive sorbitol contributes to osmotic and oxidative damage in the lens. Therefore, compounds that inhibit aldose reductase, preserve glutathione, or reduce protein glycation are mechanistically coherent candidates. Retinal findings for neem are notable but remain based on a prolonged animal experiment and require independent replication [74].

### 9.4. Diabetic Hepatopathy

Hepatoprotective claims require particularly cautious interpretation because many studies use carbon tetrachloride or galactosamine rather than a diabetes-specific model of metabolic liver disease. Changes in transaminases and antioxidant enzymes demonstrate biological activity, but they do not necessarily predict benefit in diabetic steatotic liver disease. Table 8 compares the reported preparations, models, and hepatic outcomes.

Hepatic endpoints provide a useful example of the need to separate diabetic organ injury from general toxicological hepatoprotection. CCl4 and galactosamine models demonstrate antioxidant and antifibrotic capacity, but they do not reproduce all mechanisms of metabolic dysfunction-associated steatotic liver disease in diabetes. Diabetes-specific studies should include hepatic fat, insulin signaling, inflammatory and fibrotic markers, and clinically relevant liver outcomes.

### 9.5. Diabetic Cardiomyopathy

Myocardial evidence remains sparse and is centered largely on *Gongronema latifolium*. In the primary two-week study, an 80% ethanolic leaf extract altered CK, CK-MB, LDH, and AST activities in alloxan-diabetic rats, but the responses were not dose-dependent and the investigators noted possible hepatotoxicity [83]. These enzyme changes are not specific enough to establish direct cardioprotection or to distinguish myocardial effects from skeletal-muscle or hepatic injury.

A convincing cardiomyopathy study would need ventricular structure and function, myocardial fibrosis, mitochondrial respiration, and electrophysiology, ideally with a glycemia-matched comparator. Until such evidence is available, *G. latifolium* should be described as a metabolically active experimental candidate rather than a treatment for diabetic cardiomyopathy.

The cardiomyopathy evidence is substantially narrower than the renal, neural, or ocular literature. Contemporary evaluation should combine cardiac imaging, histology, fibrosis and mitochondrial markers, electrophysiology, and functional outcomes. The absence of a dose response and of organ-specific endpoints in the available experiment limits causal interpretation [83].

## 10. Mechanistic and Botanical Integration

The literature contains a broader mechanistic inventory than can be represented by glucose-lowering outcomes alone. Reported actions include adrenergic effects, blockade of pancreatic β-cell potassium channels, inhibition of renal glucose reabsorption, reduced insulin degradation, provision of trace elements required for β-cell function, preservation of islet tissue, stimulation of hepatic glycolysis and glycogenesis, inhibition of α-amylase, α-glucosidase, and β-galactosidase, cortisol-lowering activity, and prevention of oxidative injury [1,2,6,84]. Although several of these mechanisms resemble validated drug targets, the evidence supporting each action varies markedly in quality and must be linked to the exact preparation tested.

Several mechanisms recur across otherwise unrelated plants. Polyphenols and tannins inhibit carbohydrate-hydrolyzing enzymes; *Aloe*, neem, *Pterocarpus*, and selected flavonoids influence insulin secretion or β-cell survival; and berberine-related alkaloids and other constituents affect AMPK, PI3K/AKT, and glucose transport. The same intervention may act at more than one site, particularly when a crude extract contains chemically distinct fractions [10,12,21,22]. For *Scoparia dulcis*, isolated aerial-part flavonoids and diterpenoids inhibited α-glucosidase and showed PPARγ agonistic activity in vitro [28].

This apparent multitarget activity is attractive, but it complicates causal inference. A fall in glucose can secondarily improve oxidative, inflammatory, lipid, and organ-injury markers. Mechanistic studies should therefore demonstrate exposure to the proposed active constituent and include controls capable of separating direct tissue protection from the consequences of improved glycemia.

### 10.1. Structural and Physicochemical Integration

Flavonoids comprise at least several thousand phenolic molecules occurring in fruits, vegetables, nuts, cereals, seeds, cocoa, tea, soy, wine, herbs, and beverages. Their common scaffold contains two aromatic rings connected through a three-carbon unit that commonly forms an oxygenated heterocycle. Variations in oxidation, hydroxylation, glycosylation, and attachment of the B ring generate anthocyanidins, flavan-3-ols, flavonols, flavones, flavanones, and isoflavones [21,31]. These substitutions influence intestinal absorption, phase-II metabolism, tissue distribution, redox behavior, enzyme inhibition, and interaction with signaling proteins.

Isoflavones such as genistein and daidzein are concentrated in soy and have been investigated for effects on β-cell proliferation and glucose-stimulated insulin secretion [21]. Anthocyanins—including cyanidin, delphinidin, and pelargonidin derivatives—are widely distributed in red, blue, and purple fruits and vegetables. Their dietary exposure and combined antioxidant and signaling actions make them relevant to prevention-oriented research, although food intake cannot be equated with the administration of a purified compound or concentrated extract.

Catechins and related flavan-3-ols combine radical-scavenging capacity with effects on insulin secretion, neuronal survival, vascular biology, and lens injury. Epicatechin from *Pterocarpus marsupium* was associated with increased islet cAMP, insulin release, proinsulin conversion, and cathepsin-B activity in experimental models [20]. Green-tea catechins were additionally linked to the inhibition of aldose reductase and glycated proteins in diabetic cataract models [63].

Tannins, saponins, alkaloids, glycosides, terpenoids, and polysaccharides broaden the pharmacological spectrum. Tannoid principles from *Emblica officinalis* inhibit aldose reductase, whereas arabinogalactan fractions from *Tinospora cordifolia* have shown antioxidant activity in experimental diabetes [39,53,55,75]. Protoberberines and other alkaloids, terpenoids, and polysaccharides are among the principal active groups proposed for *Tinospora*. Because these classes differ markedly in molecular size, polarity, metabolism, and bioavailability, pharmacological generalization across them is inappropriate.

The structural and physicochemical properties of phytochemicals are central to their pharmacological behavior. Flavonoid hydroxylation may increase radical-scavenging and protein-binding capacity while reducing membrane permeability, whereas glycosylation modifies aqueous solubility, intestinal uptake, and microbial metabolism [21,31]. Anthocyanins are generally present as glycosides and are sensitive to pH, light, processing, and storage. Isoflavones such as genistein and daidzein combine metabolic actions with affinity for estrogen receptors, making endocrine context important in long-term development.

Saponins are amphiphilic glycosides whose aglycone and sugar chain determine membrane, bile-acid, and absorption effects. Catechins and proanthocyanidins can influence digestive enzymes, vascular and neural oxidative stress, and protein glycation. Chlorogenic acid connects dietary exposure with intestinal glucose transport and hepatic metabolism, but is extensively metabolized. Hydrolyzable and condensed tannins may inhibit enzymes and aldose reductase, yet strong protein binding can reduce bioavailability or interfere with nutrient and drug absorption. Alkaloids range from relatively defined protoberberines to complex plant-specific mixtures and therefore require compound-level toxicological characterization.

Alkaloids range from defined agents such as berberine and *1-deoxynojirimycin* to complex, poorly resolved fractions. Polysaccharides present a different problem: molecular heterogeneity and indirect effects on the gut barrier, immunity, or microbiota often make conventional systemic pharmacokinetics difficult to interpret. Development should therefore proceed from a measured constituent or reproducible fingerprint, not from the phytochemical class alone.

### 10.2. Priority Candidates and Development Gaps

A smaller group of plants recurs across glycemic and complication-related domains. Table 9 compares these candidates while preserving species, plant part, preparation, chemical markers, evidence level, and the experiment most likely to resolve the principal development uncertainty.

## 11. Critical Appraisal and Translational Priorities

The recurring weaknesses are closely connected. Incomplete botanical identification leads to variable chemistry; variable chemistry obscures exposure; uncertain exposure weakens mechanistic interpretation; and small, short trials cannot determine durability or uncommon harms. These problems cannot be corrected statistically after a study has been completed.

A stronger program begins with an authenticated voucher specimen and a defined plant part; documents species, chemotype, cultivation, harvest, processing, solvent, extraction ratio, and storage; and sets quantitative marker, contaminant, and stability specifications. These variables can change constituent ratios and biological activity, so results from different organs, extracts, or batches should not be treated as replication. Pharmacology should use achievable free concentrations or justify local intestinal exposure and active metabolites. Clinical protocols should stabilize background medication, monitor hypoglycemia, prespecify outcomes, register the study, and use the same characterized product evaluated preclinically.

For interpretation, evidence can be viewed in four tiers: Tier 1, replicated controlled human evidence for a chemically specified product; Tier 2, one controlled human study supported by plausible chemistry, exposure, and mechanism; Tier 3, coherent animal and cellular evidence without adequate human validation; and Tier 4, ethnomedical use or screening activity without sufficient product characterization. Table 9 applies this logic to priority candidates. The hierarchy is not a formal grading instrument, but it prevents mechanistic abundance or recurrence of a plant name from being mistaken for independent clinical replication.

The literature is most convincing when independent lines of evidence meet. Digestive-enzyme inhibition, glucose transport, insulin secretion, redox regulation, and tissue protection have each been observed with more than one chemical family. Convergence, however, does not rescue a poorly characterized product. If the chemical composition changes between experiments, an apparently replicated botanical result may in fact represent several different interventions.

Clinical design should follow the proposed therapeutic role. An intestinal enzyme inhibitor may be tested through meal-related glucose excursions, whereas an insulin sensitizer requires a longer exposure and stable background treatment. Trials aimed at nephropathy, neuropathy, or ocular disease need sufficient duration and organ-specific primary outcomes. Randomization, allocation concealment, blinding, prospective registration, and active surveillance for hypoglycemia remain essential, but methodological correctness must be matched by a chemically reproducible product.

## 12. Safety and Clinical Positioning

Phytotherapy should be positioned as supervised adjunctive care or as a discovery platform, not as an unqualified replacement for insulin or validated glucose-lowering medication. Beyond short-term tolerability, development programs should evaluate durable hepatic and renal safety, cardiovascular effects, reproductive and developmental toxicity, allergy, contamination, and uncommon adverse events. Particular caution is warranted in pregnancy, childhood, frailty, advanced kidney or liver disease, and perioperative care. Products containing toxic species, contaminants, undeclared pharmaceuticals, or poorly characterized mixtures should not enter clinical research without rigorous quality control.

Medication reconciliation is essential because additive hypoglycemia may occur with insulin, sulfonylureas, metformin, or other glucose-lowering agents. Antiplatelet or anticoagulant, antihypertensive or diuretic, and lipid-lowering therapies may add pharmacodynamic risk, while CYP enzymes and intestinal or renal transporters create pharmacokinetic interaction pathways. Long-term trials should prespecify hepatic, renal, cardiovascular, and hypoglycemia monitoring and document all supplements and concomitant medicines [89,90].

## 13. Contemporary Clinical and Translational Evidence

The contemporary update focused on controlled human studies published from 2020 through July 2026 that evaluated a defined plant-derived preparation, purified fraction, or optimized derivative in type 2 diabetes. Randomized studies were prioritized, together with pharmacokinetic and interaction studies capable of clarifying why a product might succeed or fail clinically. Results were interpreted at the product level: evidence for one extract, dose, or formulation was not extrapolated to other products marketed under the same botanical name.

The clinical literature remains much smaller than the experimental literature and is heterogeneous in plant part, extraction, chemical specification, background medication, duration, and outcome selection. Particular weight was therefore given to between-group effects, clinically interpretable glycemic endpoints, adverse-event reporting, and whether product chemistry was sufficiently described to permit replication. Biomarker improvements were considered supportive rather than equivalent to the prevention of a diabetic complication. Two recent trials are particularly instructive. The 24-week ESOLED pilot trial found no significant time-by-group effect of olive leaf extract on HbA1c, whereas an eight-week trial of *Celosia argentea* seed powder reported improvements in HbA1c and quality of life in only 40 participants. The former emphasizes the value of publishing neutral findings; the latter remains hypothesis-generating because of its small sample, short duration, and limited chemical characterization [91,92].

### 13.1. Randomized and Controlled Human Evidence

Human evidence remains much narrower than the experimental literature and is strongly product-specific. Trial results cannot be generalized from a standardized extract to every powder, tea, or supplement sold under the same botanical name. The design, exposure, principal findings, and interpretive limitations of selected controlled studies are compared in Table 10.

Statistical significance was not equated with clinical importance. Interpretation emphasized between-group differences, magnitude of HbA1c change, duration, adverse events, and product reproducibility. Small samples, short follow-up, multiple outcomes, and biomarker-only endpoints reduce certainty even when nominal *p* values are significant.

Across these studies, the magnitude and credibility of benefit vary substantially. Berberine-based products and mulberry alkaloids currently possess the clearest translational logic because a chemically defined active fraction can be connected to a measurable pharmacological mechanism. Cinnamon, bitter melon, *Nigella sativa*, neem, fenugreek, and *Coccinia grandis* provide human signals, but the evidence generally remains adjunctive and product-specific. Results should not be generalized from one proprietary or standardized extract to powders, teas, tinctures, or supplements marketed under the same botanical name.

Commercial involvement was considered explicitly. The standardized fenugreek trial [41] was partially funded by Chemical Resources, a product-associated organization, and the ESOLED trial [91] reported an industry-associated research grant and product-sector donations. The former reported unusually large effects, whereas the latter produced a neutral HbA1c result. Sponsorship does not automatically invalidate a study, but it is a risk-of-bias domain; effect sizes that diverge from established clinical norms require transparent reporting and independent replication.

### 13.2. Pharmacokinetics, Formulation, and Herb–Drug Interactions

Oral exposure is a central translational constraint. Berberine illustrates the problem: extensive intestinal metabolism and transport limit systemic bioavailability, while a small crossover pharmacokinetic study suggested higher exposure with dihydroberberine than with conventional berberine. Because that study included only five men, it is hypothesis-generating rather than definitive [34]. Strategies such as salt selection, phospholipid complexes, nanoparticles, amorphous dispersions, permeability enhancers, and prodrug design may improve exposure, but enhanced bioavailability can also amplify toxicity and interaction risk. Formulation claims therefore require matched pharmacokinetic, pharmacodynamic, and safety data rather than dissolution data alone.

Experimental concentrations should be interpreted against human pharmacokinetics. Free plasma exposure—not the nominal oral dose or total concentration—should be compared with in vitro activity after accounting for protein binding, metabolism, transport, and tissue distribution. High intestinal concentrations may justify a local α-glucosidase or microbiome mechanism even when systemic exposure is low, whereas cellular effects observed only at supra-physiological concentrations cannot be assumed to mediate clinical benefit. Dose–exposure–response and active-metabolite studies are therefore required.

Pharmacodynamic interaction is particularly relevant when botanical products are added to metformin, sulfonylureas, insulin, or other glucose-lowering agents. A review of antidiabetic herb–drug interactions identified plausible modulation of drug-metabolizing enzymes, transporters, and shared glucose-lowering pathways [89]. In an Algerian survey, oral-antidiabetic-drug users were approximately twice as likely to combine herbs with conventional therapy; olive leaves and fenugreek were common, creating a credible risk of additive hypoglycemia [90]. Interaction assessment should therefore include CYP and transporter profiling, renal and hepatic function, hypoglycemia surveillance, and documentation of all supplements—not merely the prescribed drug list.

A botanical product intended for pharmaceutical development requires more than a reproducible HbA1c signal. Identity and composition must remain stable from batch to batch; contaminants, adulterants, and degradation products need explicit limits; and dose selection should be supported by pharmacokinetics or a defensible local-exposure argument. Safety assessment should include hepatic and renal function, hypoglycemia, reproductive risk where relevant, and the potential to alter drug-metabolizing enzymes or transporters.

Clinical studies should specify the background regimen and use outcomes appropriate to the proposed role. A postprandial enzyme inhibitor, an insulin sensitizer, and an organ-protective adjunct should not be evaluated as though they were interchangeable products. The comparator, duration, and primary endpoint must follow the pharmacology.

## 14. Pharmaceutical Development Priorities

The central development question is not whether a plant has ever lowered glucose in an experimental model, but whether a reproducible botanical drug, purified constituent, or optimized derivative can be manufactured, dosed, and monitored with predictable benefit–risk. On this basis, candidates can be ranked according to the convergence of chemical definition, mechanistic coherence, human evidence, and developability.

Chemically defined berberine preparations and mulberry alkaloids rich in *1-deoxynojirimycin* were prioritized because a measurable constituent or fraction, a coherent mechanism, standardized dosing, and controlled human glycemic outcomes converge. Even for these candidates, evidence is product-specific, and independent replication with comparable chemistry, longer follow-up, and active comparators remains limited. Neem, fenugreek, cinnamon, bitter melon, *Nigella sativa*, and *Coccinia grandis* occupy an intermediate position because their human signals are generally short, formulation-specific, or insufficiently replicated.

*Curcuma longa* and other anti-inflammatory or antioxidant preparations may be more useful for complication-oriented research than for large reductions in glucose. Most remaining ethnomedical extracts should still be regarded as exploratory. Their next decisive step is not another uncontrolled glucose experiment, but authentication, chemical dereplication, exposure-informed pharmacology, and a controlled proof-of-concept study.

**Figure 4:**
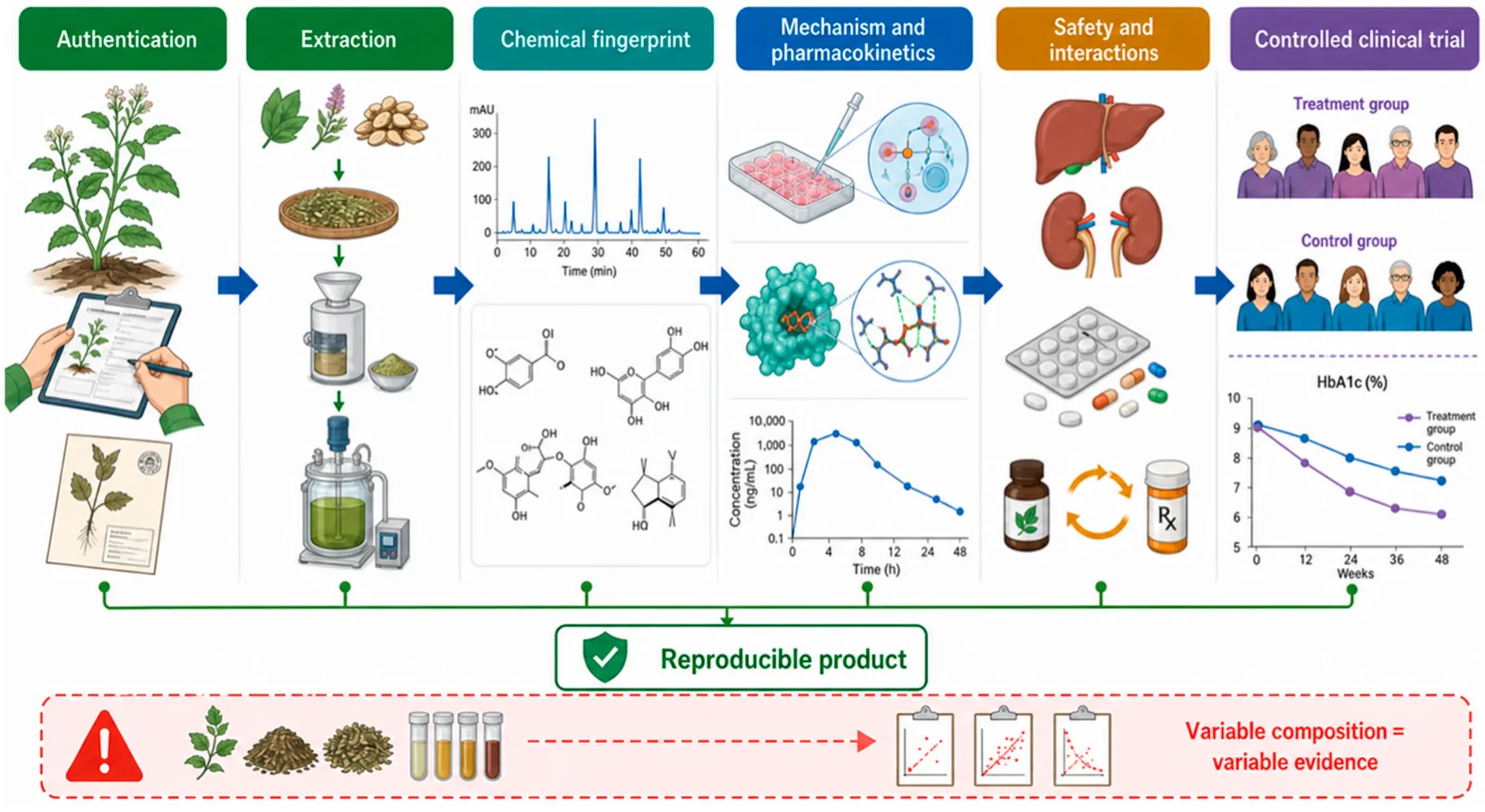
Pharmaceutical development pathway from medicinal-plant authentication to controlled clinical evaluation. Reproducible development requires authenticated raw material, a defined extraction process, chemical fingerprinting, mechanism and pharmacokinetic studies, safety and interaction assessment, and controlled clinical trials. Variable composition produces variable evidence. Created in BioRender. Varut R. (2026) https://app.biorender.com/illustrations/canvas-beta/6a623cdd32b0513368df09c9.

A candidate should advance from preclinical to clinical development only when five go/no-go criteria are met: (1) authenticated species and plant part with a reproducible chemical specification; (2) a mechanism linked to disease-relevant functional endpoints; (3) activity at pharmacokinetically achievable systemic exposure or a defensible local-exposure mechanism; (4) adequate repeat-dose toxicology and a defined herb–drug interaction plan; and (5) independent confirmation or robust internal replication followed by a prospectively registered, adequately powered trial using the identical product. Multi-omics can support responder and mechanism analyses but cannot substitute for analytical chemistry, exposure measurement, and controlled clinical design. The resulting development sequence is shown in Figure 4.

## 15. Conclusions

The evidence documents a wide antidiabetic phytopharmacology, but clinically interpretable benefit is concentrated in a small number of chemically defined products. In contemporary controlled studies, berberine-based interventions reduced HbA1c by approximately 0.7–0.99 percentage points, depending on formulation and comparator [32,33]. Standardized mulberry twig alkaloids rich in *1-deoxynojirimycin* provide a coherent intestinal α-glucosidase-inhibitory strategy with controlled human evidence [35]. These signals are promising but remain product-specific; they do not establish equivalent efficacy for all berberine or mulberry supplements.

The literature is too heterogeneous in species, plant part, extraction, chemical specification, dose, model, background therapy, duration, and outcome definition to support pooled quantitative conclusions in this narrative review. Three immediate actions are needed: publish batch-level chemical specifications linked to pharmacokinetic exposure; replicate priority products independently in adequately powered, prospectively registered trials with clinically meaningful endpoints; and build long-term safety and herb–drug interaction surveillance into development. Until then, plant-derived products should complement rather than replace evidence-based diabetes care, and claims must remain tied to the exact tested preparation.

## Figures and Tables

**Table 1:** Principal molecular and physiological targets.

Target	Proposed Action	Representative Plants/Compounds	References
*Carbohydrate digestion*	Inhibition of α-amylase and α-glucosidase; delayed glucose appearance	*Scoparia dulcis*; flavonoids; tannins; alkaloids	[10,12]
*Insulin secretion*	Stimulation of β-cell insulin synthesis and/or release; reduced insulin degradation	*Aloe barbadensis*; *Allium cepa*; *Pterocarpus marsupium*	[13,14,15]
*β-cell survival*	Antioxidant, antiapoptotic, and regenerative effects	*Azadirachta indica*; flavonoids; *Pterocarpus marsupium*	[16,17,18,19,20]
*Glucose transport*	PI3K/AKT- and AMPK-associated GLUT4 translocation	Flavonoids; *Artemisia dracunculus*; multiple extracts	[21,22]
*Hepatic metabolism*	Increased hexokinase/glucokinase and glycogenesis; reduced gluconeogenesis	*Allium cepa*; hepatoprotective extracts	[14,15,23,24,25,26,27]
*Nuclear signaling*	PPARγ agonism and improved insulin sensitivity	*Scoparia dulcis* constituents; polyphenols	[28]
*Incretin axis*	Incretin-mimetic effects and DPP-4 inhibition	Selected plant extracts and isolated phytochemicals	[29,30]
*Oxidative stress*	Radical scavenging; metal chelation; increased SOD, CAT, GPx, GST and GSH	Flavonoids; tannins; *Emblica officinalis*; *Cichorium* spp.	[12,21,23,24,26,27,31]

**Table 2:** Lead phytochemicals and marker groups relevant to pharmaceutical development.

Lead Constituent or Marker	Representative Botanical Origin	Principal Targets or Actions	Analytical/Development Value	Main Limitation
Berberine and defined derivatives	*Berberis*/*Coptis*-related botanical sources and formulated derivatives	AMPK-associated metabolic signaling; microbiome and bile-acid effects	Chemically defined lead with human efficacy and PK-development programs [32,33,34]	Low oral bioavailability; gastrointestinal adverse effects; transporter and interaction potential
1-Deoxynojirimycin-rich alkaloids	Mulberry twig alkaloid preparations	Intestinal α-glucosidase inhibition and postprandial glucose control	Quantifiable, mechanism-linked fraction suitable for standardized tablets [35]	Product-specific evidence; gastrointestinal tolerability and long-term comparative effectiveness
Quercetin and related flavonols	Fruits, vegetables, medicinal plants, *Emblica* and other taxa	Redox and inflammatory signaling; aldose-reductase inhibition	Widely used analytical marker and mechanistic probe [36]	Extensive conjugation and low free systemic exposure; findings cannot be generalized to all flavonoids
(−)-Epicatechin	*Pterocarpus marsupium* bark	Islet cAMP, insulin release, proinsulin processing, and experimental β-cell effects	Links a traditional bark preparation to a defined molecule [20]	Preclinical evidence dominates; extract–compound equivalence and human exposure remain uncertain
Genistein, daidzein, and puerarin	Soy and other isoflavone-containing plants	β-cell and insulin-signaling effects; neural and ocular protection	Chemically identifiable subclass with structure-related activity [21]	Endocrine activity, food-matrix effects, and microbiome-dependent metabolism
Gallic acid, ellagic acid, and tannoid principles	*Emblica officinalis* and polyphenol-rich fruits	Antioxidant and anti-inflammatory activity; aldose-reductase inhibition	Useful markers for tannin-rich preparations and ocular hypotheses [37,38,39]	Protein binding, variable hydrolysis, and uncertain contribution of circulating metabolites
Curcuminoids	*Curcuma longa* standardized extracts	Nrf2, NF-κB, redox-inflammatory and endothelial pathways	Standardized high-curcuminoid products have controlled human data [40]	Poor native bioavailability; formulation-specific exposure and biomarker-dominant endpoints
Furostanolic saponins	Standardized fenugreek seed extracts	Glucose and lipid regulation; possible insulin-sensitizing actions	Quantitative marker specification can distinguish proprietary extracts [41]	Large reported effects require independent replication; commercial sponsorship must be considered

**Table 3:** Major classes of antidiabetic phytochemicals and representative 2D structures retrieved from PubChem [42].

Class	Examples	Main Activities in the Evidence Base	Representative 2D Structure(s)	References
*Flavonoids*	Quercetin, rutin, kaempferol, luteolin, apigenin, baicalein	Antioxidant and anti-inflammatory actions; β-cell protection; GLUT4 modulation; aldose-reductase inhibition	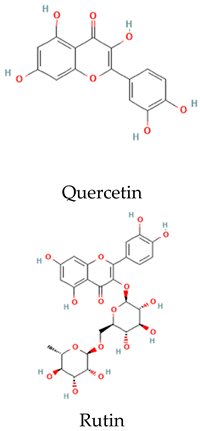	[20,21,31,36,43,44,45]
*Isoflavones*	Genistein, daidzein	β-Cell proliferation and glucose-stimulated insulin secretion	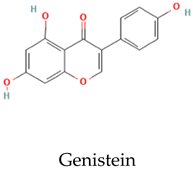	[21]
*Anthocyanins*	Cyanidin, delphinidin, pelargonidin	Antioxidant activity and modulation of glucose handling	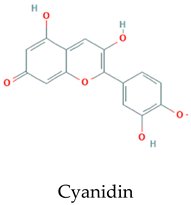	[21]
*Catechins*	Catechin, epicatechin, epigallocatechin gallate	Insulinotropic, antioxidant, neuroprotective, and anticataract effects	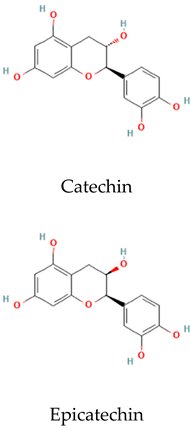	[20,21,45,46]
*Alkaloids*	Berberine and protoberberines; *Tinospora* alkaloids	Glucose uptake, enzyme inhibition, and hypoglycemic activity	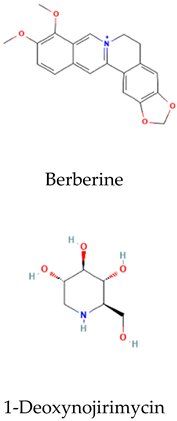	[17,29,32,34,47,48,49]
*Saponins/glycosides*	Saponins from *Berberis* and other species; diverse glycosides	Hypoglycemic, antioxidant, and possible insulinotropic effects	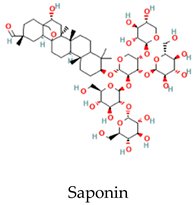	[3,50]
*Tannins/polyphenols*	Gallic acid, ellagic acid, tannoid principles	Antioxidant, neuroprotective, and aldose-reductase-inhibitory effects	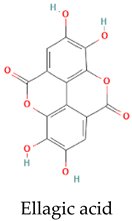	[37,38,39,44,45,51,52]
*Polysaccharides*	Arabino-galactan from *Tinospora cordifolia*	Antioxidant and immunomodulatory activity; possible complication protection	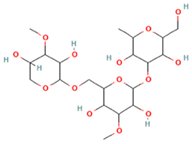	[53,54,55]
Flavonoid glycosides	Hesperidin, diosmin	Antioxidant, microvascular, and neuroprotective actions	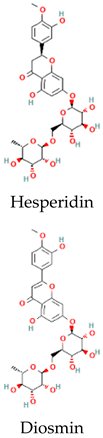	[43]
Curcuminoids	Curcumin and related curcuminoids	Nrf2/AMPK-associated antioxidant and anti-inflammatory actions	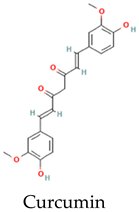	[40,56,57]

**Table 5:** Preclinical plant-derived compounds evaluated in experimental diabetic nephropathy.

Plant	Relevant Constituents/Preparation	Reported Renal or Systemic Action	Evidence Level/Model	References
Quercetin	Purified flavonol	Attenuation of albuminuria, renal dysfunction, and oxidative stress	Streptozotocin-induced diabetic nephropathy in rats	[72]
Curcumin	Purified curcuminoid	Improved albuminuria, glomerular injury, oxidative stress, and renal lipid metabolism	Type 2 diabetic nephropathy in rats; AMPK/Nrf2-associated effects	[57]
Rosmarinic acid	Isolated phenolic acid; rosemary and oregano are representative botanical sources	Reduced glomerular hypertrophy and glomerulosclerosis in experimental diabetes	Preclinical evidence summarized in a focused complication review	[63]
Naringin	Citrus flavanone glycoside	Reduced renal dysfunction, oxidative stress, and podocyte injury through NOX4-associated signaling	Streptozotocin-induced nephropathy in rats and high-glucose-exposed podocytes	[73]

**Table 6:** Preclinical plant-derived candidates evaluated for diabetic neuropathy.

Plant/Compound	Botanical Origin or Dietary Context	Reported Neuroprotective Action	Evidence Level/Model	References
*Baicalein*	*Scutellaria baicalensis* root	Improved motor/sensory conduction deficits, thermal hypoalgesia and tactile allodynia; inhibited microglial radicals and activated Nrf2-related protection	Animal/cell	[43,58]
*Chrysin*	Fruits and vegetables	Antioxidant, anti-inflammatory, antidepressant and anti-amyloidogenic effects; reduced oxidative and ER-stress-related neuronal death	Cell/preclinical	[43]
*Diosmin and hesperidin*	Citrus flavonoids	Antioxidant and microvascular neuroprotection	Preclinical review	[43]
*EGCG and proanthocyanidins*	Tea and polyphenol-rich plants	Radical scavenging and neuronal protection	Preclinical review	[43]
*Kaempferol, luteolin, myricetin, naringenin, quercetin, rutin*	Multiple plant foods	Promotion of neuronal survival and neurite growth; reduction in apoptosis, inflammation and oxidative stress	Cell/animal review	[43]
*Puerarin*	*Pueraria*-derived isoflavonoid	Neuroprotective and microvascular effects	Preclinical review	[43]
*Ellagic acid*	Berries and nuts	Antioxidant, anti-inflammatory and anti-apoptotic effects; modulation of mitochondrial dysfunction	Preclinical	[51]

**Table 7:** Preclinical plants and phytochemicals grouped by ocular target.

Candidate	Ocular Target/Mechanism	Reported Result	Evidence Level/Model	References
*Azadirachta indica*	Retinal injury; glucose and lipid control	Long-term aqueous leaf extract reversed externally visible and retinal abnormalities in STZ-diabetic rats	Animal	[74]
*Tinospora cordifolia*	Oxidative stress in retinopathy; systemic control	Polysaccharides and alkaloids linked to antioxidant and hypoglycemic effects	Preclinical	[53,54,55,75]
*Phyllanthus emblica*/*Emblica officinalis*	Aldose reductase and cataractogenesis	Fruit extract and tannoid principles delayed diabetic cataract; important aldose-reductase inhibition	Animal	[37,38,39]
*Pterocarpus marsupium*	Hyperglycemia and lens protection	Aqueous bark extract showed anticataract activity in alloxan-diabetic rats	Animal	[20]
*Green tea*	Aldose reductase, glycation, oxidative stress	Inhibited aldose reductase and glycated proteins and showed anticataract activity	Animal	[63]
*Adhatoda vasica*	Lens aldose reductase	Anticataract activity through enzyme inhibition	Preclinical	[63]
*Trigonella foenum-graecum*	Hyperglycemia and aldose reductase	Anticataract activity associated with glucose lowering and enzyme inhibition	Preclinical	[63]
*Ocimum sanctum*; *Silybum marianum*	Polyol accumulation and glutathione	Reduced polyol accumulation and increased GSH	Animal	[68,76]
*Hydrocotyl bonariensis*	Lens protein oxidation and apoptosis	Reduced protein precipitation, lipid peroxidation, apoptosis, and epithelial proliferation	Animal	[77]
*Curcumin*	Oxidative/osmotic stress and lens proteins	Improved lipid peroxidation, GSH, polyol enzymes, and protein solubility	Animal	[56]
*Aralia elata*	Aldose reductase and oxidative stress	Prevented cataractogenesis in lens culture and STZ-diabetic rats	Ex vivo/animal	[78]
Quercetin, myricetin, puerariafuran, and puerarin	Aldose reductase and lens epithelial injury	Protected the lens through enzyme inhibition and/or epithelial-cell effects	Preclinical	[45,69,79,80]

**Table 8:** Preclinical plants grouped by hepatoprotective evidence and model.

Plant	Preparation/Constituents	Reported Hepatic Effects	Evidence Level/Model	References
*Phyllanthus emblica*	*Emblica officinalis* extract; chyavanaprash	Reduced lipid peroxides and hepatic/serum enzyme abnormalities; attenuated collagen/hydroxyproline changes and fibrosis	CCl4 rat model	[23]
*Scoparia dulcis*	PDM extract; isolated scoparic acid D; aerial-part diterpenoids and flavonoids	α-Glucosidase inhibition and PPARγ agonism in vitro; insulin secretion and glucose lowering in STZ models; attenuation of CCl4-induced biochemical and histological liver injury	In vitro assays/STZ rats and islets/CCl4 mouse model	[24,28,81,82]
*Cichorium intybus*	Ethanolic whole-plant extract	Hypoglycemic and hypolipidemic effects, with modulation of hepatic glucose-6-phosphatase activity	STZ rat model	[25]
*Cichorium glandulosum*	Root extract	Reduced serum AST, ALT, and ALP and attenuated acute hepatic injury	CCl4/galactosamine hepatotoxicity in mice	[26]
*Cichorium endivia*	Aqueous suspension of leaf powder, 500 mg/kg for six weeks	Reduced serum AST, ALT, ALP, and LDH; decreased hepatic SOD and increased GST and GSH. Changes in hepatic CAT, GPx, and MDA were not significant with *C. endivia* leaf powder	STZ-diabetic rat model	[27]

**Table 9:** Comparative profiles of high-priority antidiabetic plants.

Plant	Part/Preparation and Major Constituents	Glycemic Evidence	Complication-Related Evidence	Critical Development Need
*Aloe barbadensis*/*A. vera*	Leaf extract and bitter principle; chemically distinct fractions	Chronic leaf treatment and acute/chronic bitter-principle activity in alloxan models	Predominantly indirect protection through improved metabolism	Define anthraquinone-free composition, active markers, laxative burden, and clinical dose [13]
*Allium cepa*	Onion juice, dried-powder fractions, S-methyl cysteine sulfoxide	Glucose lowering with changes in hepatic hexokinase and glucose-6-phosphatase	Hypolipidemic and antioxidant actions may reduce vascular risk	Standardize organosulfur chemistry and evaluate antiplatelet/drug interactions [14,15,62]
*Azadirachta indica*	Leaves, twigs, seeds, bark and aqueous/alcoholic extracts; bitter limonoid-related principles	Insulinotropic, peripheral-utilization and possible β-cell-preserving effects	Experimental retinal recovery, lipid improvement, anti-inflammatory and vascular signals	Separate plant-part products; reproductive, hepatic and interaction safety; independent trials [16,61,74,85,86]
*Tinospora cordifolia*	Stem/whole-plant preparations; protoberberines, other alkaloids, terpenoids, polysaccharides	Hypoglycemic and antioxidant activity in experimental diabetes	Arabinogalactan and chemically complex extracts have been associated with retinal and neural protection [53,54,55,75]	Chemotype control and rigorous hepatic/immunological safety assessment [48,49,53,54,55,75,87]
*Phyllanthus emblica*/*Emblica officinalis*	Fruit; gallic/ellagic acids, tannins, rutin, quercetin, vitamins and minerals	Acute glucose reduction in normal and alloxan-diabetic rats	Aldose-reductase inhibition, delayed cataract, antioxidant and antifibrotic hepatic effects	Identify tannoid markers and distinguish diabetic hepatoprotection from CCl4 protection [37,38,39]
*Pterocarpus marsupium*	Bark aqueous/alcoholic fractions and isolated (−)-epicatechin	Glucose tolerance, cAMP-linked insulin secretion, proinsulin conversion and β-cell repair signals	Anti-cataract and triglyceride/insulin-resistance effects [88]	Compare standardized bark extract with purified epicatechin at human-relevant exposure [20]
*Scoparia dulcis*	Aerial-part flavonoids and diterpenoids; isolated scoparic acid D; PDM extract [24,28,81,82]	α-Glucosidase inhibition and PPARγ agonism in vitro; insulin secretion and glucose lowering in STZ rats and isolated islets [28,82]	Free-radical-scavenging activity and attenuation of CCl4-induced biochemical and histological liver injury in mice [24]	Standardize the extract and marker panel; validate hepatic effects in diabetes-specific liver models
*Cichorium* species	*C. intybus* ethanolic whole-plant extract; *C. glandulosum* root extract; *C. endivia* leaf-powder suspension	*C. intybus* showed hypoglycemic and hypolipidemic effects in STZ rats [25]	*C. glandulosum* attenuated CCl4/galactosamine hepatotoxicity [26]. *C. endivia* improved serum AST, ALT, ALP, and LDH and selected hepatic antioxidant indices in STZ-diabetic rats [27]	Keep species and preparation claims separate; test metabolic steatosis, fibrosis, pharmacokinetics, and human exposure
*Gongronema latifolium*	Leaf aqueous/ethanolic extracts; β-sitosterol, lupenyl/pregnane esters, glycosides, oils and saponins	Experimental hypoglycemic, hypolipidemic and antioxidant activity	Short-duration biochemical evidence; no dose-dependent response and possible hepatotoxicity in the primary study [83]	Use cardiac imaging, histology, fibrosis, mitochondrial, and functional endpoints; establish organ-specific safety

**Table 10:** Selected controlled clinical studies, statistical findings, and qualitative assessment of clinical relevance.

Intervention and Product	Design and Sample	Dose and Duration	Principal Findings	Safety and Critical Appraisal
Berberine; microbiome-oriented preparation	Randomized, double-blind, four-arm trial; 409 newly diagnosed adults	Berberine, probiotics, combination, or placebo; 12 weeks	HbA1c fell by approximately 0.99 percentage points with berberine versus 0.59 with placebo; microbiome and bile-acid changes were implicated [32].	Clinical relevance: modest-to-moderate and product-specific. Relatively strong contemporary dataset, but gastrointestinal adverse events were more frequent and replication across products remains necessary.
Berberine ursodeoxycholate	Randomized placebo-controlled trial; 113 adults with T2DM	500 or 1000 mg twice daily; 12 weeks	Dose-dependent placebo-adjusted HbA1c reductions of approximately 0.4 and 0.7 percentage points [33].	Clinical relevance: dose-dependent and moderate at the higher dose. Early efficacy is interpretable for this defined derivative, but duration was short and long-term comparative safety is unknown.
*Azadirachta indica* aqueous leaf/twig extract	Randomized, double-blind, placebo-controlled trial; 80 metformin-treated adults	125, 250, or 500 mg twice daily; 12 weeks	Improvements were reported in fasting and postprandial glucose, HbA1c, insulin resistance, endothelial, inflammatory, platelet, and lipid outcomes [93].	Clinical relevance: uncertain. Multiple outcomes and a modest sample increase false-positive concern; independent replication and fuller product characterization are priorities.
*Momordica charantia* standardized product	Randomized placebo-controlled trial; 90 participants in the efficacy analysis	12 weeks	Fasting glucose decreased, but HbA1c did not differ significantly from the placebo [94].	Clinical relevance: no demonstrated durable HbA1c benefit. The neutral HbA1c result weighs against claims of established glycemic control.
Standardized fenugreek seed extract	Randomized, double-blind, placebo-controlled add-on trial; 81 completers	1000 mg/day; >45% furostanolic saponins; 12 weeks	Large improvements in glycemic indices were reported [41].	Clinical relevance: potentially large but unconfirmed. Chemical standardization is a strength; unusual effect size and commercial involvement require independent confirmation.
Fenugreek dry extract	Randomized placebo-controlled trial; 54 adults with T2DM	335 mg three times daily; 8 weeks	No significant between-group benefit was found for glycemic indices, insulin resistance, triglycerides, or pro-oxidant–antioxidant balance; HDL-C improved versus placebo [95].	Clinical relevance: no demonstrated glycemic benefit. The largely neutral between-group result is more informative than within-group changes; the study was small and short.
Cinnamon preparation	Triple-blind randomized placebo-controlled trial; 160 adults	3 g/day; 90 days	Placebo-adjusted reductions were approximately 0.2 percentage points for HbA1c and 0.55 mmol/L for fasting glucose [96].	Clinical relevance: modest. Species, extraction, and coumarin content are essential to benefit–risk interpretation.
*Nigella sativa* oil	Double-blind randomized placebo-controlled trial; 50 adults	1000 mg/day; 8 weeks	Fasting glucose, lipid indices, hs-CRP, and malondialdehyde improved [97].	Clinical relevance: uncertain. The metabolic and inflammatory signal is promising, but sample size and exposure were insufficient for durable efficacy or uncommon harms.
*Coccinia grandis* standardized leaf preparation	Double-blind randomized placebo-controlled trial in newly diagnosed T2DM	Product-specific regimen reported by the trial; short-term treatment	HbA1c and related metabolic outcomes improved [98].	Clinical relevance: uncertain and product-specific. Multicenter replication and enough chemical detail to establish product comparability are required.
Mulberry twig alkaloids rich in *1-deoxynojirimycin*	Multicenter randomized, double-blind, placebo-controlled trial	Standardized alkaloid tablet	Improved glycemic control through an intestinal α-glucosidase-inhibitory strategy [35].	Clinical relevance: promising and product-specific. Mechanism and chemical definition are comparatively strong; long-term comparative safety and effectiveness remain unresolved.
*Curcuma longa* extract	Randomized, double-blind, placebo-controlled trial; 76 adults	1200 mg/day; 3 months	Endothelial glycocalyx, redox-inflammatory biomarkers, and neuropathy symptoms improved [40].	Clinical relevance: supportive for biomarkers and symptoms, not hard complication endpoints. The controlled study was short.
*Gymnema*-containing combination	Randomized controlled trial; 75 adults	Inositols, α-lactalbumin, *Gymnema sylvestre*, and zinc	No significant glucose-parameter advantage; lipid benefits were reported [99].	Clinical relevance: no demonstrated glycemic benefit attributable to *Gymnema*. The multi-ingredient formulation prevents component-level attribution.
*Olea europaea* leaf extract (ESOLED)	Pilot randomized placebo-controlled trial; 31 adults	Product-specific capsules; 24 weeks	No significant time-by-group interaction for HbA1c, insulin sensitivity, diabetes-related distress, or quality of life [91].	Clinical relevance: no demonstrated benefit. The trial was well-tolerated but underpowered, and the time-by-group HbA1c result was neutral.
*Celosia argentea* seed powder	Randomized, double-blind, placebo-controlled trial; 40 adults	4 g/day as adjunctive therapy; 8 weeks	HbA1c and quality-of-life outcomes improved; all participants completed follow-up [92].	Clinical relevance: uncertain. The trial was very small, single-center, short, and used a minimally characterized powder; independent between-group replication is required.

## Data Availability

The files and data supporting this study are retained in the physical and electronic archive of the University of Medicine and Pharmacy of Craiova and are available from the corresponding author upon reasonable request. The summarized data generated or analyzed during the study are included in this article; further inquiries may be directed to the corresponding author.
